# Ubiquitin Specific Peptidase 22 Regulates Histone H2B Mono-Ubiquitination and Exhibits Both Oncogenic and Tumor Suppressor Roles in Cancer

**DOI:** 10.3390/cancers9120167

**Published:** 2017-12-06

**Authors:** Lucile M-P. Jeusset, Kirk J. McManus

**Affiliations:** 1 Department of Biochemistry & Medical Genetics, University of Manitoba, Winnipeg, MB R3E 0V9, Canada; jeussetl@myumanitoba.ca; 2 Research Institute in Oncology and Hematology, CancerCare Manitoba, Winnipeg, MB R3E 0V9, Canada

**Keywords:** USP22, H2Bub1, cancer, SAGA complex, DUBm, genome stability, DNA double-strand break repair, chromosome instability, cell cycle regulation, precision medicine

## Abstract

Ubiquitin-Specific Peptidase 22 (USP22) is a ubiquitin hydrolase, notably catalyzing the removal of the mono-ubiquitin moiety from histone H2B (H2Bub1). Frequent overexpression of *USP22* has been observed in various cancer types and is associated with poor patient prognosis. Multiple mechanisms have been identified to explain how *USP22* overexpression contributes to cancer progression, and thus, USP22 has been proposed as a novel drug target in cancer. However, gene re-sequencing data from numerous cancer types show that *USP22* expression is frequently diminished, suggesting it may also harbor tumor suppressor-like properties. This review will examine the current state of knowledge on *USP22* expression in cancers, describe its impact on H2Bub1 abundance and present the mechanisms through which altered *USP22* expression may contribute to oncogenesis, including an emerging role for USP22 in the maintenance of genome stability in cancer. Clarifying the impact aberrant *USP22* expression and abnormal H2Bub1 levels have in oncogenesis is critical before precision medicine therapies can be developed that either directly target *USP22* overexpression or exploit the loss of *USP22* expression in cancer cells.

## 1. Introduction

Histones are small nuclear proteins (11–15 kDa) and key effectors of DNA compaction. Histones share a common structure comprised of a central globular domain along with carboxy- and amino-terminal tails. The globular domain is necessary for histone-histone and histone-DNA interactions. Two copies of each of the core histones H2A, H2B, H3 and H4 assemble into an octamer around which approximately 146 base pairs of DNA are wrapped, forming the fundamental unit of DNA compaction called the nucleosome [1]. The globular domains and the histone tails, which extend from the nucleosome, are the substrates for a vast array of Post-Translational Modifications (PTMs), including acetylation, phosphorylation, methylation and ubiquitination [2]. Conceptually, these PTMs can be viewed as modulating the balance between accessibility and compaction of the chromatin fiber, which regulates essential processes such as transcription, DNA damage repair, chromosome compaction and segregation [2]. Accordingly, the misregulation of histone PTMs is a driving force in oncogenesis, and the proteins responsible for the addition (writers), interpretation (readers) and removal (erasers) of specific histone PTMs are frequently altered in many cancer types [3,4,5,6,7].

USP22 is a histone-modifying enzyme whose predominant function is the removal of the mono-ubiquitin moiety from lysine 120 of H2B (H2Bub1) [8]; however, USP22 has also been reported to target the mono-ubiquitin moiety on lysine 119 of H2A (H2Aub1) [9] and some non-histone substrates (see Section 7, Section 8 and Section 9). As *USP22* overexpression occurs in multiple cancer types, its possible oncogenic roles and potential as a new therapeutic target in cancer are areas of active research [10,11,12,13,14,15,16,17,18,19]. However, emerging data indicate that *USP22* expression is more frequently downregulated in many cancer types, suggesting it may also harbor tumor-suppressor properties. Accordingly, this review will present the current knowledge regarding *USP22* expression in cancer and describe how both increased and diminished *USP22* expression may promote oncogenesis. In particular, we will present the oncogenic properties associated with *USP22* overexpression in transcription activation, cell death and cell cycle progression and then will describe the putative tumor suppressor role of USP22 in the preservation of genome stability. Characterizing the oncogenic and tumor suppressor functions of USP22 and its impact on H2Bub1 is essential for the development of precision medicine therapies tailored to the specific genetic context of patients with altered *USP22* expression.

## 2. Ubiquitination Is a Multi-Functional PTM

**Figure 1:**
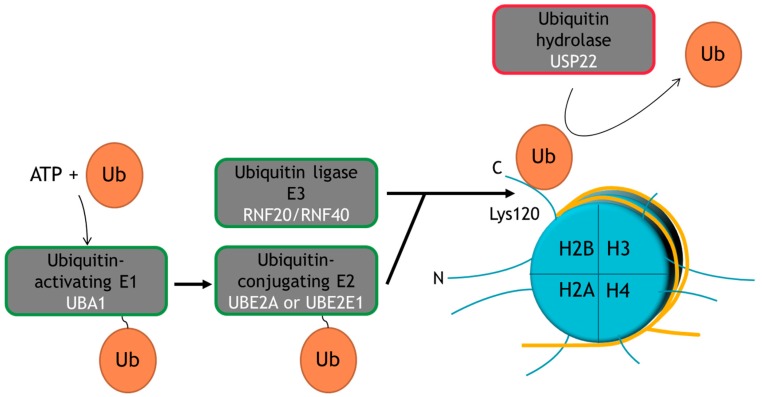
Schematic depiction of the enzymatic machinery catalyzing the addition and removal of H2Bub1. Ubiquitination requires cooperation between a ubiquitin-activating enzyme (E1), a ubiquitin-conjugating enzyme (E2) and a ubiquitin ligase (E3). The ubiquitin (Ub) can be removed from the target protein by ubiquitin hydrolases, like USP22. For illustrative purposes, the enzymes regulating H2Bub1 abundance are indicated.

Ubiquitin is a 76-amino acid residue polypeptide (8.5 kDa) consisting of a globular domain and a flexible, six-amino acid residue C-terminal tail that terminates with a glycine residue. Ubiquitination forms an isopeptide bond between this terminal glycine residue and the ε-amino group of a lysine residue contained within the target protein [20,21]. As illustrated in Figure 1, protein ubiquitination is completed through the successive action of three enzymes. First, a ubiquitin-activating enzyme (E1) is loaded with a ubiquitin moiety in an ATP-dependent reaction. The ubiquitin moiety is subsequently transferred to a ubiquitin-conjugating enzyme (E2), before it is finally transferred from the E2 to the target protein by an ubiquitin ligase (E3) [20,22]. Substrates may be mono-ubiquitinated, in which case only a single ubiquitin moiety is covalently attached, or poly-ubiquitinated, in which case chains of two to as many as ten ubiquitin moieties are covalently attached to one of six lysine residues within the ubiquitin molecule [20,21]. E3 enzymes are primarily responsible for defining substrate specificity [20], and the interplay between E3 enzymes and ubiquitin hydrolases ensures the dynamic regulation of ubiquitination. For example, the abundance of H2Bub1 is regulated by the opposing activity of the E3 complex RNF20/RNF40 and the ubiquitin hydrolase USP22 (Figure 1).

The effects of ubiquitination on target proteins depend on the length of the ubiquitin chain and the topology of the ubiquitin linkage (reviewed in [21]). Briefly, linear Lys48-conjugated poly-ubiquitin chains generally target a protein for proteasomal degradation, whereas Lys63-conjugated chains and mono-ubiquitination mainly regulate protein localization within the cell, induce conformational changes or regulate the interaction with other proteins [21]. For example, H2Bub1, which is located at the interface between nucleosomes, disrupts chromatin compaction to promote a more open and accessible chromatin fiber [23], which has important implications for the regulation of transcription (detailed in Section 6) and DNA damage repair (detailed in Section 9). On the contrary, H2Aub1 has no such effect on chromatin compaction, but is instead responsible for the recruitment of the polycomb transcription repression complex PRC2 [24,25].

## 3. USP22 Is an Evolutionarily-Conserved Ubiquitin Hydrolase within the SAGA (Spt-Ada-Gcn5 acetyltransferase) Complex

**Figure 2:**
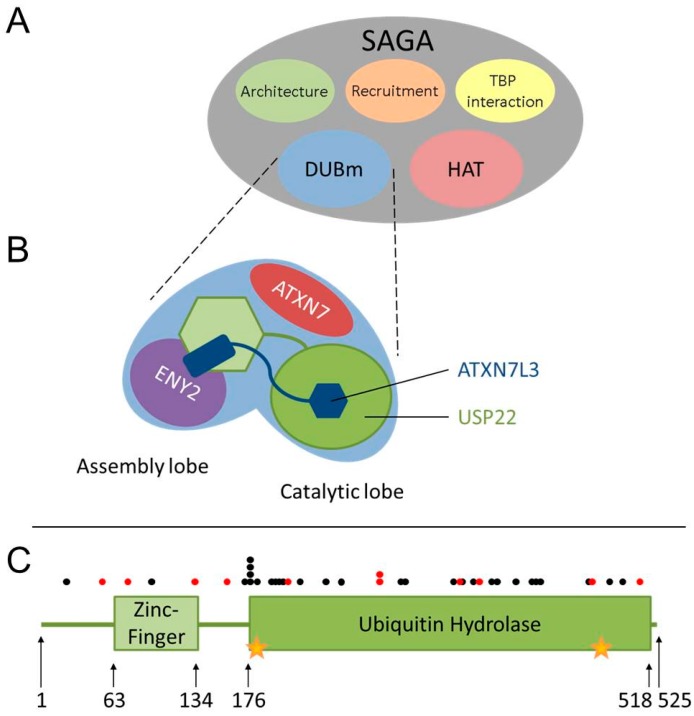
The SAGA complex ubiquitin hydrolase, *USP22,* is frequently mutated in cancer. (**A**) SAGA is a multi-module protein complex with two catalytically-active modules: the histone acetyl transferase (HAT) module and the deubiquitinase module (DUBm). TBP: TATA-binding protein. (**B**) The DUBm assembly lobe is formed by ENY2 and the USP22 N-terminal zinc-finger motif (green hexagon) structured around the N-terminal helix of ATXN7L3 (blue rectangle), while ATXN7 anchors the DUBm to the rest of SAGA. The catalytic lobe is comprised of the C-terminal ubiquitin hydrolase domain of USP22 (green circle) and the N-terminal zinc-finger domain of ATNX7L3 (blue hexagon). (**C**) Schematic depiction of the protein domains within USP22 along with the positions (circles) of cancer-associated alterations. Arrows indicate the starting positions (amino acid residues) of the protein and its domains. Stars identify the position of the two key evolutionarily-conserved catalytic residues, Cys185 and His479. Circles indicate the positions of amino acid substitutions (black) and non-sense/frame-shift mutations (red) [37].

In humans, *USP22* maps to chromosome 17p11.2. *USP22* is comprised of 13 exons encoding a 1578 nucleotide-long mRNA, which translated produces a protein comprised of 525 amino acids (~60 kDa) [26,27]. *USP22* is ubiquitously expressed in adult mammalian tissues and is predominantly enriched within the nucleus [10,26,28]. USP22 is an evolutionarily-conserved ubiquitin hydrolase, both in sequence and function (Table 1). As indicated above, the predominant function of USP22 and its orthologs, Nonstop (*Drosophila melanogaster*) and Ubp8 (*Saccharomyces cerevisiae*), is the removal of H2Bub1 [8,29,30,31], but it also removes H2Aub1 [9,29] and modulates the level of poly-ubiquitination of several non-histone substrates (see Section 7, Section 8 and Section 9). USP22 is comprised of two major domains including the N-terminal zinc-finger motif (aa 63–134) and the catalytically-active C-terminal ubiquitin hydrolase domain (aa 176–518) (Figure 2). USP22 is a cysteine protease, and its ubiquitin hydrolysis activity is catalyzed by the two conserved residues Cys185 and His479 [32,33].

The deubiquitinating activity of USP22 mandates USP22 be assembled into the Spt-Ada-Gcn5 acetyltransferase (SAGA) complex. SAGA is a large (~2 MDa) multi-module complex (reviewed in [34]) with two main catalytic activities, namely acetylation of histone H3 and deubiquitination of histones H2A and H2B (Figure 2A). Within SAGA, USP22 complexes with ATXN7L3, ATXN7 and ENY2 to form the Deubiquitinating module (DUBm) that contains an assembly lobe and a catalytic lobe (Figure 2B). The assembly lobe is comprised of ENY2, the N-terminal helix of ATXN7L3 and the N-terminal zinc-finger motif of USP22 [8,29,32,35], while the catalytic lobe contains the N-terminal zinc-finger domain of ATXN7L3 and the C-terminal ubiquitin hydrolase domain of USP22 [8,29,32]. ATXN7 functions by anchoring the DUBm within the SAGA complex, and DUBm assembly is necessary to maintain USP22 in a catalytically-active conformation [35,36]. In addition, ATXN7L3 is critical for directing the DUBm substrate specificity towards H2Bub1 [35]. 

Although the depletion of any DUBm component is predicted to prevent USP22 function and increase global H2Bub1 levels, adult mouse models with constitutively-reduced *USP22* expression lack global increases in H2Bub1 levels [38]. Further, Atanassov et al. [39] showed that in two human cell lines, depletion of ATXN7L3 or ENY2 had a greater impact on global H2Bub1 levels than USP22 depletion, as additional ubiquitin hydrolases (USP27X and USP51) can compete with USP22 to interact with ATXN7L3 and ENY2 and remove H2Bub1. Interestingly however, this compensatory mechanism does not function in all cellular processes in which *USP22*-mediated H2Bub1 regulation is involved, including transcriptional activation and DNA damage repair [8,29,40] (see Section 6 and Section 9). These contrasting observations highlight the need for further characterization of the relationship between the DUBm and H2Bub1 to identify the cellular processes and cellular contexts (i.e., cell type, cell cycle stage, cellular stress response) in which USP22 depletion is not compensated by additional ubiquitin hydrolases to reveal precisely when and where USP22 activity is critical for accurate H2Bub1 regulation.

## 4. *USP22* Expression Is Frequently Altered in Cancer

Altered *USP22* expression was first implicated in cancer in 2005 when its upregulation was identified as part of an 11-gene expression signature, termed “death-from-cancer”, that defined primary tumors with stem cell-like expression profiles. This gene signature identifies a small subset of patients with early-stage prostate, breast or lung carcinoma who are more likely to face shorter intervals to recurrence, metastatic dissemination and death after therapy [41,42]. This initial study spurred widespread interest in the potential role and prognostic significance of *USP22* overexpression in various cancer types. Subsequently, immunohistochemistry (IHC) and RT-qPCR-based studies found USP22 abundance to be higher in cancerous than normal samples and associated with worse patients outcomes in multiple cancer types including colorectal, breast, esophageal, ovarian, pancreatic and stomach cancers [10,11,12,13,14,15,16,17,18,19]. For example, *USP22* expression was significantly increased in primary colorectal carcinoma tissues relative to matched normal adjacent tissues at the protein and mRNA level [10,15]. In addition, positive USP22 IHC labeling in primary colorectal carcinoma was associated with a reduced five-year disease-free survival rate (26%) compared to USP22-negative cancers (71%) [15]. In pancreatic ductal adenocarcinoma, strong USP22 IHC labeling was more prevalent in primary cancer tissues than normal tissues and was associated with shorter overall patient survival than weak or absent USP22 labeling [16]. In stomach cancer, USP22 abundance increased from normal tissue to primary carcinoma to lymph node metastasis and was also associated with shorter patient survival (26 vs. 59 months disease-specific survival for USP22-positive vs. -negative primary carcinoma) [12].

Although collectively, the IHC-based studies detailed above suggest that *USP22* is predominantly overexpressed in cancer, the technical limitations of the approaches employed do not preclude diminished *USP22* expression from also contributing to oncogenesis. First, the above studies failed to provide information regarding antibody validation, giving rise to concerns over antibody specificity. Secondly, few studies employed appropriate control tissue samples (i.e., normal matched patient samples) to establish normal/baseline USP22 levels in a given patient, thus preventing an accurate comparison. Furthermore, it is currently unclear in which contexts additional deubiquitinating enzymes may have compensated for the loss of USP22 (see Section 3). Since few studies concomitantly evaluated USP22 levels and the functional readout of its activity (i.e., changes in H2Bub1 and/or additional substrates), it is unclear the extent to which aberrant *USP22* expression adversely impacted its established substrate targets and contributed to oncogenesis.

**Figure 3:**
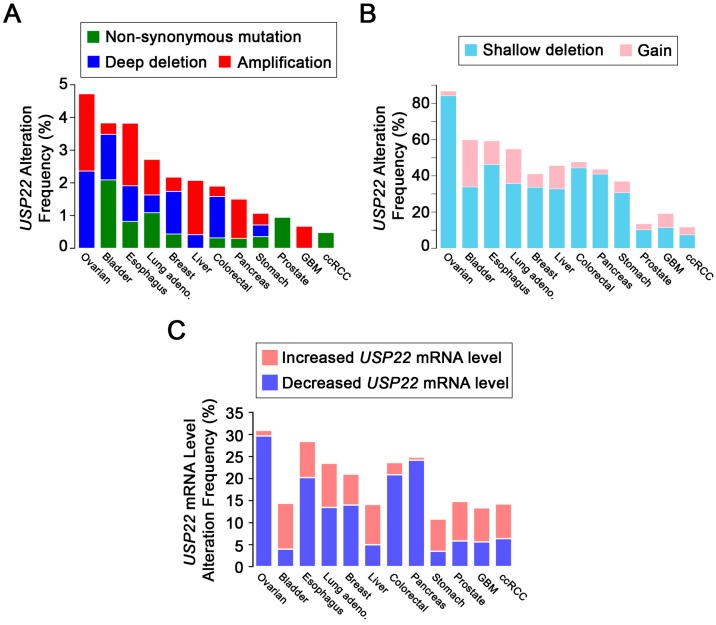
Frequencies of *USP22* alterations in cancers evaluated by The Cancer Genome Atlas (TCGA) [37]. (**A**) Mutations, deep deletions (i.e., heterozygous loss) and amplifications are observed at low frequency. (**B**) Shallow deletions (i.e., heterozygous loss) and gains of *USP22* are frequent events in many cancer types. (**C**) Frequencies of cases for each cancer type with increases (pink) or decreases (blue) in *USP22* mRNA expression (z-score ± 1.5). Lung adeno: Lung adenocarcinoma; GBM: Glioblastoma Multiforme; ccRCC: clear cell Renal Cell Carcinoma. All data are from TCGA [37,43,44,45,46,47,48,49,50,51].

The high prevalence of *USP22* overexpression in cancer is also challenged by the results of many additional genome-wide studies. Indeed, gene sequencing and mRNA expression data reveal that *USP22* is more frequently deleted, mutated or underexpressed than amplified or overexpressed in those same cancer types for which IHC studies found *USP22* to be overexpressed. At the gene level, data from The Cancer Genome Atlas (TCGA) indicate that in many cancer types, *USP22* is more frequently lost (homozygous or heterozygous loss) than gained [37]. In fact, in a survey of TCGA gene re-sequencing data from the 12 most deadly cancer types in North America, each cancer exhibits a greater frequency of *USP22* copy number losses than gains (Figure 3). While homozygous deletion of *USP22* occurs in 0–4% of most cancers (Figure 3A), shallow deletions (i.e., heterozygous loss) are the most prevalent forms of copy number alterations (Figure 3B). Notably, shallow deletions of *USP22* occur frequently in ovarian (84% of cases), esophagus (46%), colorectal (44%), pancreatic (41%), lung adenocarcinoma (38%), breast (34%) and stomach (31%) cancers. In contrast, low-level gains and amplification of *USP22* are collectively observed in only 2–20% of cases from the same cancer types (Figure 3B). Further, RNA-sequencing data also show that *USP22* mRNA expression is more frequently reduced in ovarian, esophagus, breast, colorectal, pancreatic and stomach cancers (Figure 3C) [37]. Accordingly, these data suggest that *USP22* may be a haplo-insufficient tumor suppressor gene, whose diminished expression may also contribute to cancer development.

The differences between TCGA sequencing data and IHC-based studies regarding the level of *USP22* expression may stem from various parameters, in addition to the technical limitations of the IHC studies discussed above. First, while IHC evaluates protein abundance, sequencing approaches evaluate gene copy numbers and mRNA levels. Importantly, post-translational regulatory mechanisms can stabilize a protein that appears downregulated at the mRNA level, by increasing protein half-life. If such a mechanism regulates USP22 in cancer, it may potentially explain why *USP22* overexpression is more frequently observed via IHC than mRNA sequencing. Alternatively, the results may simply reflect underlying technical differences in sample selection and preparation, as well as variations in the patient cohorts. For instance, as several IHC studies found USP22 abundance to vary with disease stage [11,12], differences in the distribution of disease stages within patient cohorts may bias study results toward increased or reduced *USP22* expression.

## 5. The Challenges of Evaluating USP22 Activity in Cancer

Although DNA/RNA sequencing and IHC studies provide insight into *USP22* expression in cancer, none are sufficient to accurately evaluate altered USP22 activity. Indeed, USP22 activity depends on its incorporation into the DUBm and thus will be impacted by the three remaining DUBm members, ATXN7, ATXN7L3 and ENY2. Therefore, it is important to examine these genes and proteins for altered expression, as well. For example, at the mRNA level in ovarian cancer, *USP22* is downregulated in 20% of cases and upregulated in 2.5% (Figure 3C); while collectively, at least one DUBm gene (*USP22*, *ATXN7L3*, *ATXN7* or *ENY2*) is either downregulated in 47% of cases, or upregulated in 21% of cases (RNA-sequencing TCGA data) [37,51].

USP22 function is not only impacted by aberrant DUBm member expression, but also by specific mutations. Whole-exome sequencing studies show that non-synonymous *USP22* mutations typically occur in 1–4% of any given cancer type (Figure 3A) [37]. Over 80 distinct non-synonymous *USP22* mutations have been reported in multiple cancer types in TCGA [37]. Of these, 28 encode single amino acid substitutions that are predicted to impair USP22 function by at least two of three common online prediction algorithms (PolyPhen-2 [52], SIFT [53] and Mutation Assessor [54], Figure 2C). Importantly, expression of USP22 mutants with reduced activity may mimic hypomorphic *USP22* expression. In addition, biochemical data suggest that certain *USP22* mutations may exert dominant negative effects. For example, Atanassov et al. [39] determined that expression of a catalytically-dead form of USP22 induced a greater increase of H2Bub1 levels in embryonic kidney cells than *USP22* silencing alone, thus demonstrating that certain *USP22* mutations impair the removal of H2Bub1.

To bypass the complexity of evaluating the expression and possible mutations of each DUBm gene, H2Bub1 abundance may be employed as a proxy for the balance between the activity of the H2Bub1 writer (RNF20/40) and its eraser (DUBm or additional ubiquitin hydrolases). In this regard, several IHC-based studies have begun to evaluate H2Bub1 abundance in lung, breast, ovarian and colorectal cancers [11,55,56,57,58], but the frequency of abnormal H2Bub1 levels remains unclear. For example, in colorectal cancer, estimates of the proportion of cases with absent or weak H2Bub1 staining relative to non-cancerous controls vary widely from 43–86% [11,55,57]. The underlying reasons accounting for these disparate results are unclear, but are likely technical in nature, and may include issues associated with identifying appropriate controls to establish baseline levels of H2Bub1 (i.e., stromal cells vs. normal adjacent tissues).

Collectively, the data discussed above highlight a need for further rigorous evaluation of *USP22* expression and activity in cancer and, in particular, its impact on H2Bub1. Two subsets of cancers appear to coexist; those with increased *USP22* expression and those with reduced expression, both of which may contribute to oncogenesis. This suggests that *USP22* expression may be tightly regulated and that deviations, either as increases or decreases, have functional implications for cancer development and progression. Accordingly, subsequent sections will explore how processes normally regulated by USP22 are impacted by aberrant *USP22* expression and promote oncogenesis.

## 6. USP22 Is a Transcriptional Activator

As indicated above, USP22 regulates the abundance of H2Aub1 and H2Bub1 to promote gene expression and is therefore considered a transcriptional activator. H2Aub1, which is catalyzed by the Polycomb Repressive Complex 1 (PRC1), is enriched within the promoter regions of polycomb target genes, promotes the recruitment of PRC2 repressive complex and promotes transcriptional repression [25,59,60]. Therefore, USP22 is proposed to promote transcription of polycomb targets by reversing H2A ubiquitination [9]. Unlike H2Aub1, H2Bub1 is enriched within transcribed genes [36,61] and is proposed to promote transcription by disrupting local chromatin structure, thus increasing accessibility for the transcription machinery [23,62]. In addition, H2Bub1 cooperates with the FACT (Facilitate Chromatin Transcription) complex to enable the displacement of the H2A/H2B dimer, promoting the progression of the RNA polymerase II through the nucleosomal barrier. H2Bub1 is therefore critical for the transition of the RNA polymerase II from transcription initiation to elongation and is associated with increases in transcription efficiency and transcript length [63,64]. However, studies in yeast have shown that both initial H2B ubiquitination and its subsequent removal by Ubp8 (USP22 ortholog) are necessary to achieve the highest expression of SAGA-regulated genes [65]. More specifically, H2Bub1 removal enables the recruitment of an RNA polymerase II kinase, which promotes transcription progression [66]. In humans, the DUBm was shown to be active on H2Bub1 within the gene body of all actively transcribed genes and thus act as a global transcriptional activator [36,61].

*USP22* expression is necessary for the active transcription of target genes of several oncogenic transcription factors. Zhang et al. [8] reported that *USP22* expression is required for active expression of c-Myc target genes. In a c-Myc-dependent manner, USP22 is recruited to the promoter of these genes, where it presumably progresses, along with the SAGA complex, throughout the transcribed region of the genes [8]. Importantly, *USP22* silencing was shown to inhibit c-Myc-driven transformation of human fibroblasts, indicating that *USP22* expression supports the oncogenic role of c-Myc [8]. In prostate cancer, USP22 was found to promote expression of genes co-regulated by c-Myc and the androgen receptor [67]. In addition, *USP22* expression was shown to increase the abundance of c-Myc and the androgen receptor itself [16,67,68]. Indeed, USP22 upregulates the abundance of the androgen receptor by protecting it from proteasomal degradation through deubiquitination [67]. c-Myc abundance is also indirectly increased by the activity of USP22 on its substrate SIRT1, a NAD-dependent protein deacetylase [69] (see Section 7), but this is not observed in all cell types [70]. Thus, USP22 upregulation has widely been proposed to support oncogenic-driven proliferation of cancer cells by increasing the abundance of oncogenic transcription factors and the expression of their target genes [10,67,69]; however, it should be noted that the role of USP22 as transcriptional activator is not limited to the expression of oncogene targets, as USP22 is also required for active transcription of multiple target genes of TP53, a major tumor suppressor [8]. Therefore, USP22 appears to be a global transcriptional activator, which may be recruited by various transcription factors. In cancer cells, whether altered *USP22* expression (i.e., increased or decreased) promotes the expression of oncogenes or tumor suppressors is likely dependent on the cellular context, as c-Myc amplification and TP53 status will impact the effect of USP22 on transcription and oncogenesis.

## 7. USP22 Regulates Cell Death to Enhance Resistance to Chemotherapy

Multiple studies show that *USP22* silencing increases apoptosis in both mouse and human embryonic fibroblasts, as well as in multiple cancer cell lines, including colorectal and brain glioma cell lines [71,72,73]. Conversely, *USP22* overexpression in HeLa cells was shown to attenuate apoptosis induced by trichostatin A (histone deacetylase inhibitor) treatment [74]. USP22 is proposed to primarily regulate apoptosis by modulating the abundance of its substrate SIRT1, a protein deacetylase. USP22 catalyzes the removal of poly-ubiquitin chains from SIRT1 to prevent its degradation and effectively increase its abundance [73]. In turn, SIRT1 deacetylates TP53 to inhibit transcriptional activation of TP53 target genes [73]. Although Lin et al. [73] reported that the USP22/SIRT1/TP53 regulatory pathway prevents DNA-damage-induced apoptosis in embryonic kidney and colorectal cancer cell lines, this observation was disputed by Armour et al. [75] who showed USP22 depletion had no effect on SIRT1 stability or TP53 acetylation status within the same cell lines. Nonetheless, upregulation of the USP22/SIRT1/TP53 regulatory pathway has also been reported in acute myeloid leukemia stem cells harboring an internal tandem duplication mutation of the tyrosine kinase gene, *FLT3*, which confers resistance to common tyrosine kinase inhibitors employed in the clinic. In acute myeloid leukemia cells, the *FLT3* mutation was associated with increases in c-Myc activity, USP22 levels and SIRT1 abundance and correlated with TP53 target gene repression [69]. Further, SIRT1 was shown to stabilize c-Myc through deacetylation in a positive feedback loop that inhibited apoptosis, resulting in a decrease in sensitivity to tyrosine kinase inhibitors [69]. Hence, in multiple cancer cell lines, increased *USP22* expression attenuates apoptosis and promotes treatment resistance.

Beyond regulating apoptosis, the USP22/SIRT1 pathway may also modulate autophagy. Autophagy is the controlled destruction and recycling of intra-cellular damaged proteins or organelles to support cellular metabolism [76]. In cancer, sustained autophagy can lead to cell death, but it can also promote resistance to cellular stress and tumor survival [77]. In both hepatocellular and pancreatic cancers, increased autophagy is proposed to support oncogenesis and increase resistance to treatment [77,78,79]. Interestingly, two autophagy effectors, ATG5 and ATG7, are substrates of SIRT1 [80] and thus may be impacted indirectly by altered USP22 activity. In hepatocellular carcinoma cell lines, the long non-coding RNA, lncHULC, promotes *USP22* expression, which increases SIRT1 abundance and thus increases deacetylation and activation of ATG5 and ATG7, to induce autophagy. This regulatory pathway is upregulated and reduces sensitivity to treatments with conventional chemotherapies, like oxaliplatin and 5-fluorouracil, in cell lines and xenograft models of hepatocellular carcinoma [80]. Higher levels of autophagy are also observed in pancreatic ductal adenocarcinoma tissues where *USP22* is reportedly overexpressed relative to normal tissues [14]. In addition, in a pancreatic cancer cell line (Panc-1), *USP22* overexpression stimulates autophagy through the ERK1/2 pathway and hereby promotes resistance to gemcitabine treatment [14]. *USP22* upregulation may thus inhibit apoptosis and stimulate autophagy in response to treatment with DNA damaging agents or targeted inhibitors to promote resistance to chemotherapy in cancer patients.

## 8. USP22 Modulates Cell Cycle Progression

Altered *USP22* expression is also linked to an additional cancer hallmark [6], namely the alteration of cell cycle progression and checkpoints. Several studies have shown that reduced *USP22* expression correlates with a G1 accumulation in normal human fibroblasts [8] and in lung, colorectal and breast cancer cell lines [8,68,70,72]. Conversely, *USP22* overexpression facilitates the G1/S transition and leads to an S-phase accumulation in pancreatic cancer cell lines [16]. Multiple mechanisms have been proposed to explain the role of USP22 in the control of the G1/S transition. For example, USP22 was shown to catalyze the removal of a Lys63 poly-ubiquitin chain from the transcription regulator Far Upstream Binding Protein 1 (FBP1). This poly-ubiquitin chain inhibits binding of FBP1 to its target sequence and notably prevents FBP1 from repressing the expression of its target p21, an inhibitor of the cyclin/Cyclin-Dependent Kinase (CDK) complexes required for G1/S transition [70]. Hence *USP22* silencing reduces the capacity of FBP1 to repress p21 (independently of TP53 status), which in turn inhibits CDKs to prevent the G1/S transition and resulting in G1 accumulation [70]. In pancreatic cancer cell lines, an alternative mechanism was reported, whereby USP22 was found to modulate the β-catenin/Wnt signaling and thus increase the abundance of FoxM1, a transcription factor that normally represses the expression of two CDK inhibitors, p21 and p27 [16]. Hence, *USP22* overexpression was associated with reduced p21 and p27 levels and increased abundance of Cyclin D1, CDK4 and CDK6, which collectively form a complex that promotes G1 progression [16].

Although several studies indicate that USP22 depletion results in an accumulation of cells in G1, a few studies indicate that USP22 may also have a role in G2/M progression. For example, *USP22* silencing in brain glioma cell lines was associated with reduced CDK1 and Cyclin B1 expression, two proteins required for mitosis initiation and progression, and induced a G2/M arrest [71]. While the underlying mechanism accounting for the reduction in CDK1 and Cyclin B1 was not explored, Cyclin B1 was identified as a USP22 substrate in an independent study that determined Cyclin B1 deubiquitination by USP22 prevented its degradation [81]. This regulatory mechanism suggests that overexpression of *USP22* may facilitate mitotic entry via abnormal Cyclin B1 upregulation.

Although currently unknown, the reason for this apparent dichotomy between studies reporting that USP22 depletion induces the accumulation of cells in either G1 or G2/M may be differences in the genetic context of the cell lines in which these experiments were performed. Indeed, alteration of the cell cycle checkpoints is a frequent feature of cancer cells [6,82], which may modulate the effects of *USP22* overexpression and silencing in those cellular models. Highlighting the impact of cellular context, an additional study reported that diminished *USP22* expression had little to no effect on cell cycle progression in Human Embryonic Kidney cells (HEK293) [39]. Nevertheless, significant evidence is accumulating that indicates *USP22* expression promotes cell cycle progression in certain cancer cell lines and that *USP22* overexpression may enhance proliferation of cancer cells by facilitating premature transition through various cell cycle stages.

## 9. Diminished *USP22* Expression Compromises Genomic Stability

USP22 regulates essential cellular processes such as transcription, cell death and cell cycle progression, and its overexpression may alter these processes in such a way as to promote oncogenesis; however, *USP22* deletions and diminished *USP22* mRNA levels are frequently observed in many cancer types (see Section 4). Therefore, reduced *USP22* expression cannot systematically induce cell cycle arrest and hinder cancer progression, as suggested above. In fact, emerging evidence indicates that diminished *USP22* expression may also contribute to oncogenesis by promoting genome instability or the accumulation of genomic alterations including point mutations, copy number variations and chromosomal rearrangements. This aberrant phenotype, observed in virtually all cancer types, is now widely regarded as an ‘enabling characteristic’ of cancer [6,83,84]. Indeed, genome instability drives the accumulation of subsequent pathologic mutations, which enable the acquisition of additional cancer hallmarks that confer growth advantages to the cells. Notably, genome instability can arise from defects in DNA damage repair and telomere maintenance pathways [6,83]. In addition, a prominent form of genome instability termed chromosome instability, which is characterized by an increase in the rate at which whole chromosomes or large chromosome fragments are gained or lost, can be caused by chromatin compaction defects and chromosome segregation errors [85,86,87,88]. Recent emerging data now implicate USP22 and H2Bub1 as key regulators in many of these processes, and thus, hypomorphic *USP22* expression may contribute to the development of genomic instability and promote cancer progression.

Dynamic regulation of H2Bub1 abundance is critical for efficient DNA Double-Strand Break (DSB) repair through both the non-homologous end-joining and the homologous recombination repair pathways [89,90]. Indeed, the RNF20/40 complex is recruited to DSB sites where it catalyzes a local increase in H2Bub1 levels, enabling the recruitment of both non-homologous end-joining and homologous recombination repair proteins [89,90]. Importantly, a recent study shows that the subsequent removal of H2Bub1 by USP22 is critical for DSB repair. In the mouse B cell line, CH12, USP22 and the DUBm are necessary to resolve DSBs that arise naturally as a result of class switch recombination. This pathway, which generates antibody diversity, involves the introduction of a DSB in the gene encoding the heavy chain of an immunoglobulin, which is subsequently resolved through non-homologous end-joining to produce a gene that codes for a different class of heavy chain [40]. In this process, USP22 activity and H2Bub1 removal are required for efficient formation of DNA damage-induced γH2AX foci, whereas USP22 depletion impairs both non-homologous end-joining and homologous recombination repair [40]. If the function of USP22 in DSB repair is conserved in other cell types, then the loss of USP22 may prevent correct DSB repair and promote the accumulation of pathologic mutations underlying the development of cancer.

In yeast, dynamic regulation of H2Bub1 is also required during transcription-coupled repair, a process that resolves RNA Pol II stalling at DNA damage sites. RNA Pol II stalling is followed by rapid H2B deubiquitination by the two ubiquitin hydrolases Ubp8 (yeast ortholog of USP22) and Ubp10 (likely yeast ortholog of USP36). Failure to remove H2Bub1 in the double mutant (*ubp8Δubp10Δ*) decreased transcription-coupled repair efficiency, possibly by preventing eviction of the H2A/H2B dimer and thus the ability of repair proteins to access the damage site [91]. Although it has not been evaluated whether this role of Ubp8/USP22 is conserved in humans, a very rapid decrease in H2Bub1 following RNA Pol II stalling does occur in human cells [91]. Thus, this is a mechanism through which diminished USP22 expression may underlie genome instability and promote oncogenesis.

**Figure 4:**
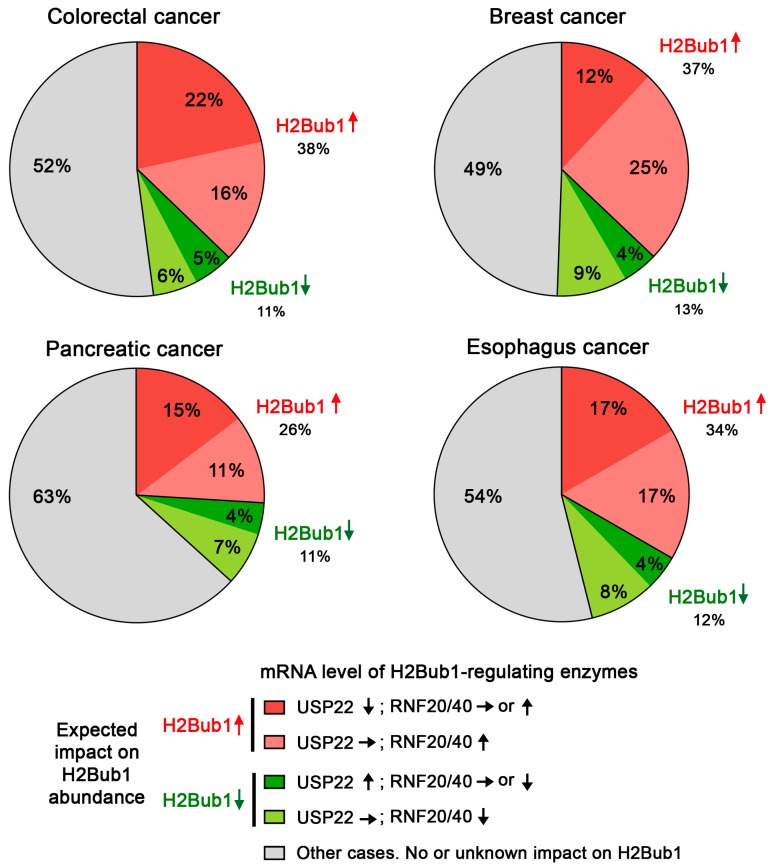
The H2Bub1 ubiquitination/deubiquitination machinery is frequently misregulated in cancer. The pie charts present the frequencies of mRNA alterations (z-score ± 1.5) of key enzymes impacting the global abundance of H2Bub1 in four cancer types. Conceptually, H2Bub1 abundance is predicted to increase when *USP22* expression is diminished (red), or *RNF20*/*RNF40* expression is increased (pink), whereas H2Bub1 levels are predicted to decrease when *USP22* expression is increased (dark green), or *RNF20*/*RNF40* expression is diminished (light green). No overt changes in H2Bub1 levels are expected when *USP22* and *RNF20/RNF40* expression is normal, or when *USP22* and *RNF20/40* expression levels are concomitantly increased, or decreased within the same tumor (grey). All data from TCGA [37,47,48,51].

As detailed above, H2Bub1 abundance varies locally in response to DNA damage (i.e., at the level of the gene), but it also exhibits global temporal changes in abundance that are cell cycle-associated. Present within interphase nuclei, H2Bub1 is rapidly removed during the early stages of mitosis (prophase and prometaphase) and remains virtually undetectable until the end of mitosis, before increasing in G1 [92]. In agreement with these temporal dynamics, USP22 abundance is lowest in G1, rises through S phase and peaks during G2/M [81]. In addition, reports indicate that USP22 is capable of rapidly depleting global H2Bub1 levels throughout the genome [29,36,61]. These observations implicate USP22 as the major deubiquitinating enzyme responsible for the rapid removal of H2Bub1 at the onset of mitosis and suggest that this process may be critical for mitotic fidelity. Interestingly, biophysical studies have shown that H2Bub1 disrupts higher-order chromatin compaction, presumably by impeding nucleosome stacking [23]. Since ubiquitin is a large PTM (8.5 kDa) relative to the size of histones (11–15 kDa) and H2Bub1 is located at the interface between nucleosomes, H2Bub1 may physically impair chromatin condensation through steric hindrance. Specific interactions between the ubiquitin moiety and the nucleosome surface may also play a role [23]. Importantly, higher-order chromatin compaction is critical for mitotic fidelity, and impaired chromatin compaction is associated with chromosome segregation defects during mitosis [86,87,93]. Therefore, H2Bub1 may represent a physical barrier during mitosis, which must be removed for proper chromosome compaction and segregation to occur. Accordingly, impaired DUBm activity, preventing timely H2Bub1 removal during mitosis, is expected to negatively impact chromosome compaction and segregation and induce chromosome instability. In support of this possibility, knockout of the DUBm components *Sgf11* or *Sgf73* (orthologs of *ATXN7L3* and *ATXN7*) in *S. cerevisiae* induces chromosome instability [94,95]. Since genes associated with the maintenance of chromosome stability are frequently conserved from yeast to humans [94,95], it is predicted that diminished *ATXN7L3*, *ATXN7, ENY2* or *USP22* expression will induce chromosome instability in humans. In addition, upregulation of the E3 ubiquitin ligases RNF20 and RNF40 required for the mono-ubiquitination of H2B is also predicted to induce chromosome instability, by interfering with the timely removal of H2Bub1 at the onset of mitosis. Remarkably, diminished expression of *USP22* and/or overexpression of *RNF20/40* at the mRNA level is observed in 38% of primary tumors in colorectal adenocarcinoma [37,48] (Figure 4), a cancer type characterized by a very high prevalence of chromosome instability (up to 85% of cases) [96,97,98,99]. Similarly, diminished *USP22* expression and/or *RNF20/40* overexpression is observed in 37% of breast cancers and 34% of esophagus cancers [37,47]. Thus, multiple lines of evidence suggest that USP22-mediated removal of H2Bub1 in early mitosis may exhibit a critical role in mitotic fidelity.

Aberrant maintenance of telomeres is also associated with chromosome instability and is a frequent feature of cancer cells. For example, telomere elongation contributes to the enhanced replicative potential of cancer cells [6]. In addition, alteration of the Shelterin complex, which normally ensures recognition and protection of the telomeres within a cell, can promote chromosomes end-to-end fusions and result in significant structural chromosome instability [6,100]. TRF1 is a component of the Shelterin complex and negatively regulates telomere length to prevent uncontrolled telomere elongation [101]. In mouse embryonic fibroblasts, USP22 was found to catalyze the removal of poly-ubiquitin from TRF1, thereby protecting TRF1 from proteasomal degradation and increasing its stability. Consequently, prolonged depletion of USP22 or ATXN7 decreased TRF1 abundance and compromised the activity of the Shelterin complex, resulting in an increased frequency of telomere dysfunction-induced foci and telomere elongation [102]. Moreover, end-to-end fusions were observed when the SAGA complex was altered in mouse fibroblasts [102]. Thus, telomere destabilization is another mechanism through which loss of *USP22* can promote chromosome instability.

## 10. Summary and Conclusions

While it is clear that *USP22* is overexpressed in various cancer types and may promote oncogenesis by altering gene expression, cell death and cell cycle progression, emerging evidence suggests that *USP22* also harbors tumor suppressor-like properties. Remarkably, reduced *USP22* expression occurs more frequently than overexpression in many cancer types suggesting diminished expression and function may also be oncogenic. By impacting H2Bub1 levels, USP22 partakes in multiple pathways required for the maintenance of genome stability, and *USP22* expression may be required to prevent aberrant events underlying genome instability. Hence, accurate regulation of *USP22* expression and function, and by extension H2Bub1 abundance, appears critical as both hypermorphic and hypomorphic USP22 activity are associated with aberrant phenotypes known to promote cancer progression.

To clarify the roles of USP22 and its main target H2Bub1 in cancers, it will be critical to assess the activity of the proteins responsible for both the addition and removal of the ubiquitin, which include not only USP22, but also the other DUBm members ATXN7, ATXN7L3 and ENY2, as well as RNF20 and RNF40. In addition, as USP22 is proposed to modulate the ubiquitination level of an ever-growing number of substrates in addition to H2B, further studies are required to functionally dissect the roles that each of these modifications have in oncogenesis. These efforts will be critical to uncover the specific contexts in which either the oncogene or tumor suppressor-like properties of USP22 are relevant, to identify instances when direct USP22 targeting may be therapeutically beneficial or when therapies that exploit reduced *USP22* expression may be appropriate. Such an understanding is vitally important so that the appropriate precision medicine strategy can be applied to the right patient.

## Figures and Tables

**Table 1:** USP22 is an evolutionarily-conserved histone deubiquitinase enzyme.

Protein	Accession Number	Species	Length ^A^	Identity ^B^ (%)	Similarity ^C^ (%)	E-Value ^D^
USP22	NP_056091.1	*Homo sapiens*	525	N/A	N/A	N/A
USP22	NP_001004143.2	*Mus musculus*	525	98	99	0.0
USP22	XP_021335835.1	*Danio rerio*	506	93	97	0.0
USP22	NP_001085912.1	*Xenopus laevis*	523	92	95	0.0
Nonstop	NP_524140.2	*Drosophila melanogaster*	496	51	65	0.0
Ubp8	NP_592992.1	*Schizosaccharomyces pombe*	449	32	46	5e-70
Ubp8	NP_013950.1	*Saccharomyces cerevisiae*	471	31	46	8e-63

^A^ Overall amino acid length; ^B^ Percentage of identical amino acids identified by BLASTp between query protein and the human USP22 protein sequence; ^C^ Percentage of similar amino acids identified by BLASTp between query protein and the human USP22 protein sequence; ^D^ As determined by BLASTp.
